# Genetic Analysis of African Swine Fever Virus From the 2018 Outbreak in South-Eastern Burundi

**DOI:** 10.3389/fvets.2020.578474

**Published:** 2020-11-05

**Authors:** Jean N. Hakizimana, Lionel Nyabongo, Jean B. Ntirandekura, Clara Yona, Désiré Ntakirutimana, Olivier Kamana, Hans Nauwynck, Gerald Misinzo

**Affiliations:** ^1^SACIDS Africa Centre of Excellence for Infectious Diseases, SACIDS Foundation for One Health, Sokoine University of Agriculture, Morogoro, Tanzania; ^2^Department of Veterinary Microbiology, Parasitology and Biotechnology, College of Veterinary Medicine and Biomedical Sciences, Sokoine University of Agriculture, Morogoro, Tanzania; ^3^National Veterinary Laboratory of Burundi, Bujumbura, Burundi; ^4^Department of Animal Health and Productions, University of Burundi, Bujumbura, Burundi; ^5^Department of Biosciences, Solomon Mahlangu College of Science and Education, Sokoine University of Agriculture, Morogoro, Tanzania; ^6^Department of Food Science and Technology, College of Agriculture, Animal Sciences and Veterinary Medicine, University of Rwanda, Busogo, Rwanda; ^7^Department of Applied Research and Development and Foresight Incubation, National Industrial Research and Development Agency, Kigali, Rwanda; ^8^Laboratory of Virology, Faculty of Veterinary Medicine, Ghent University, Merelbeke, Belgium

**Keywords:** African swine fever, *Asfarviridae*, Burundi, domestic pigs, genotyping

## Abstract

African swine fever (ASF) is a contagious viral disease that causes high mortality, approaching 100%, in domestic pigs and wild boars. The disease has neither a cure nor a vaccine, and it is caused by an ASF virus (ASFV), the only member of the family *Asfarviridae*, genus *Asfivirus*, and the only known DNA arbovirus. Twenty-four genotypes of ASFV have been described to date, and all of them have been described in Africa. ASF is endemic in Burundi, and several outbreaks have been reported in the country; the disease continues to economically impact on small-scale farmers. This study aimed at genetic characterization of ASFV that caused an ASF outbreak in the Rutana region, Burundi, in the year 2018. Tissue samples from domestic pigs that died as a result of a severe hemorrhagic disease were collected in order to confirm the disease using polymerase chain reaction (PCR) and to conduct partial genome sequencing. Nucleotide sequences were obtained for the *B646L* (p72) gene, the intergenic fragment between the *I73R* and *I329L* genes, and the central variable region (CVR) of the *B602L* gene. Phylogenetic analysis of the Burundian 2018 ASFV grouped the virus within *B646L* (p72) genotype X and clustered together with those reported during the 1984 and 1990 outbreaks in Burundi with high nucleotide identity to some ASFV strains previously reported in neighboring East African countries, indicating a regional distribution of this ASFV genotype. Analysis of the intergenic fragment between *I73R* and *I329L* genes showed that the Burundian 2018 ASFV described in this study lacked a 32–base pair (bp) fragment present in the reference genotype X strain, Kenya 1950. In addition, the strain described in this study had the signature AAABNAABA at the CVR (*B602L*) gene and showed 100% amino acid sequence identity to viruses responsible for recent ASF outbreaks in the region. The virus described in this study showed high genetic similarities with ASFV strains previously described in domestic pigs, wild suids, and soft ticks in East African countries, indicating a possible common wild source and continuous circulation in domestic pigs in the region.

## Introduction

African swine fever (ASF) is a contagious and fatal viral disease of domestic pigs and wild boar (1, 2). It is caused by the ASF virus (ASFV), the only member of the family *Asfarviridae*, genus *Asfivirus* (3), and the only known DNA arbovirus. Twenty-four (I to XXIV) genotypes of ASFV have been described to date based on nucleotide sequencing of the *B646L* gene encoding for the p72 protein (4–6), and all of them have been described in Africa (2). Depending on the virus strain, the ASFV genomes vary in length from about 170 to 193 kilobase pairs (Kbp) and contain between 151 and 167 open reading frames with a conserved central region and variable termini (7). Depending on the ASFV strain, morbidities and mortalities can reach 100%, making ASF the most serious constraint to domestic pig production, food and nutritional security, and livelihood of small-scale farmers in Africa (8). ASF has neither a cure nor a vaccine, and its effective control relies on quarantine, stamping out, and strict biosecurity measures (9, 10). ASF is endemic in many African countries south of the Sahara and in Sardinia (Italy), and in recent years, it has spread beyond its traditional geographical boundaries to the Caucasus region, the European Union, and Asia (11–14). The recent spread to China, which is the major pork-producing country, is threatening global food security (15, 16). The epidemiology of ASF is complex, transmission is direct and vector-borne, and the disease has well-recognized sylvatic and domestic cycles (17). In Eastern and Southern Africa, ASFV is maintained in a sylvatic cycle between warthogs (*Phacochoerus africanus)* and soft argasid ticks of the *Ornithodoros moubata* complex (18). Warthogs and bushpigs (*Potamochoerus* spp.) are the natural hosts of ASFV that are persistently infected with no obvious clinical disease, and soft ticks of the genus *Ornithodoros* are vectors for transmission of ASFV from the sylvatic to the domestic cycle (19). Wild natural hosts of ASFV have been reported to be present in the Kibira and Ruvubu National Parks of Burundi (20), but their role in the maintenance and transmission of the virus in the country is not known. In the domestic cycle, two transmission patterns are recognized, namely, a tick-to-pig cycle that involves soft ticks inhabiting pig shelters and an exclusively pig-to-pig cycle. Once introduced into domestic pig populations, the virus can be transmitted between domestic pigs mainly by ingestion of contaminated feeds and direct contact between infected and susceptible pigs (21).

In Eastern and Southern Africa, some ASFV genotypes are country specific, while others have a transboundary distribution (22). In Burundi, strains of ASFV described from the outbreaks of 1984 and 1990 belong to *B646L* (p72) genotype X (21). Genotype X is one of the predominant genotypes in East African countries including Tanzania, Kenya, and Uganda (23–25). Despite the regular ASF outbreak reports in domestic pigs in Burundi, molecular characterization of the causative viruses has been limited. For instance, the currently available ASFV strains genetically characterized from Burundi were collected more than two decades ago. In August 2018, an outbreak of a hemorrhagic and fatal disease affecting domestic pigs suspected to be ASF occurred in the Rutana region in South-Eastern Burundi. This study describes the confirmation and molecular characterization of the 2018 outbreak of ASFV in South-Eastern Burundi based on partial amplification and nucleotide sequencing of the *B646L* (p72) gene, the tandem repeat sequence (TRS) located between the *I73R* and *I329L* genes, and the central variable region (CVR) of the *B602L* gene.

## Materials and Methods

### Study Area, Sampling, and Sample Processing

**Figure 1:**
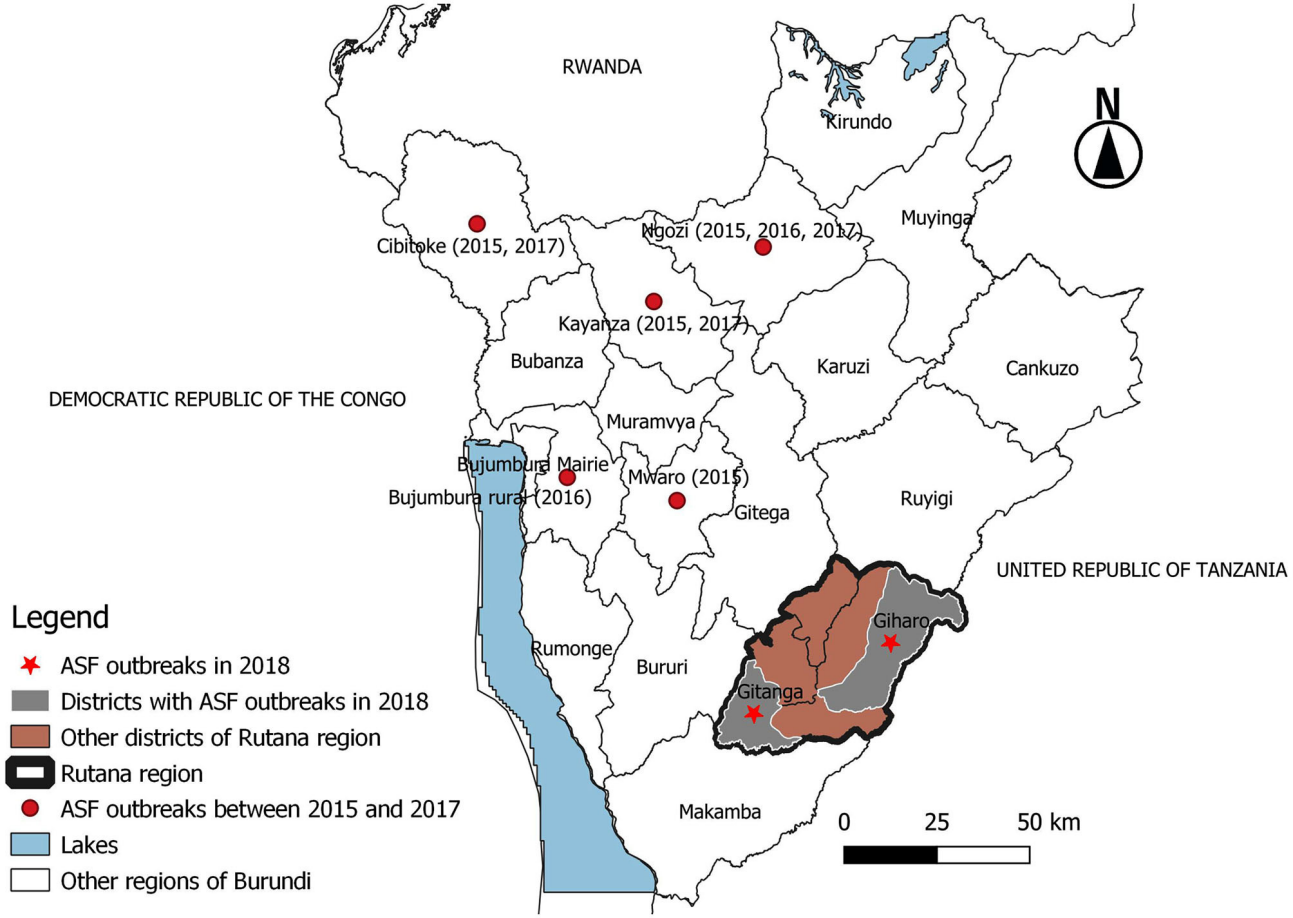
Map of Burundi showing African swine fever outbreaks between 2015 and 2018. The outbreaks between 2015 and 2017 occurred in regions located in the northern part of Burundi bordering Rwanda and western regions bordering the Democratic Republic of the Congo. The 2018 outbreak occurred in the Giharo and Gitanga districts of the Rutana region bordering Tanzania. Source: National Veterinary Laboratory of Burundi, Burundi.

An outbreak of a hemorrhagic disease associated with high mortalities in domestic pigs occurred in South-Eastern Burundi in August 2018. The disease started in Mutwana village in the Giharo district of the Rutana region in South-Eastern Burundi (Figure 1). The number of domestic pigs that died during the outbreak was recorded from Rutana Region Livestock Office records. Tissues (lung, spleen, and liver) were collected from three domestic pigs that naturally died from the disease. Each tissue (lung, spleen, and liver) was aseptically collected into a separate tube. Samples were chilled on ice and transported to the laboratory. In the laboratory, 1 g from each of the tissue samples was separately placed into a sterile petri dish and chopped using a sterile scalpel blade in the presence of 10 mL sterile phosphate-buffered saline (PBS). Afterward, homogenized tissue samples were centrifuged at 6,000 g for 5 min, and the supernatants, aliquoted into cryovials before cryopreservation at −80°C until DNA extraction.

### DNA Extraction

Frozen aliquots of lung, liver, and spleen homogenates were allowed to thaw, and DNA was extracted directly from 150 μL of homogenized tissue samples using a QiaAmp nucleic acid extraction kit (Qiagen, Hilden, Germany), following the manufacturer's instructions. Each extraction yielded 50 μL of DNA whose quantity and purity were determined by a nanodrop spectrophotometer (Biochrom, Cambridge, England) before being stored at −20°C until nucleotide amplification by polymerase chain reaction (PCR).

### Amplification of ASFV DNA

The disease confirmation was carried out by PCR using ASF diagnostic primers PPA1 and PPA2 as previously described by Agüero et al. (26). Amplification for partial nucleotide sequencing of ASFV DNA was conducted using primers that target (i) the variable 3′-end of the *B646L* gene encoding the major capsid protein p72 using primers p72D and p72U (27), (ii) a TRS located between the *I73R* and *I329L* genes using primers ECO1A and ECO1B (28), and (iii) the CVR of the *B602L* gene using the ORF9L-F and ORF9L-R primer pair (21, 29). The amplification conditions used in the present study were similar to those previously described (21, 26–29). All nucleotide amplifications were performed using AccuPower PCR premix (Bioneer, Daejeon, Republic of Korea) on a GeneAmp 9700 PCR system (Applied Biosystems, Foster City, CA). Afterward, the electrophoretic separation of amplicons was conducted on 1.5% agarose gel mixed with GelRed nucleic acid stain (Phenix Research Products, Candler, NC) against a 1 Kbp molecular weight marker (Promega, Madison, WI, USA) before visualization and imaging using a Gel Doc^TM^ EZ Imager agarose gel imaging system (Bio-Rad, Hercules, CA).

### ASFV Partial Genome Nucleotide Sequencing

PCR products from *B646L* (p72), TRS, and CVR were subjected to automated dideoxynucleotide cycle sequencing using a Big Dye Terminator Cycle sequencing kit V3.1 (Applied Biosystem, Foster City, CA) using primers: p72D, p72U, ECO1A, ECO1B, ORF9L-F, and ORF9L-R. Products from the cycle sequencing reaction were purified by ethanol precipitation and separated by capillary gel electrophoresis on an ABI 3730xl DNA analyzer (Applied Biosystems, Foster City, CA). Chromatograms for both the forward and the reverse primer reactions were checked for quality using Sequence Scanner v2.0 software (Applied Biosystems, Foster City, CA). The forward nucleotide sequence and the reverse complement nucleotide sequence from the reverse primer were subjected to pairwise alignment in Bioedit v7.2.5 (Ibis Biosciences, Carlsbad, CA) in order to obtain a single consensus nucleotide sequence delimited by the forward and reverse primers. In addition to the Burundian 2018 ASFV, the TRS between the *I73R* and *I329L* genes of the Tanzanian ASFV strains TAN/13/Arusha, TAN/16/Babati, and TAN/16/Ngara was amplified and sequenced in this study. The CVR of the *B602L* gene was amplified and sequenced for TAN/16/Ngara in the present study.

### Phylogenetic Analysis of ASFV *B646L* (p72), TRS, and CVR

The nucleotide sequences of *B646L* (p72), TRS, and CVR from the 2018 ASFV that caused an outbreak in South-Eastern Burundi were submitted to GenBank and assigned accession numbers (Tables 1, 2). The similarity search of the obtained nucleotide sequences against other ASFV sequences available at GenBank was performed using BLASTn (version 2.8.1+). The nucleotide sequence of *B646L* (p72) of the Burundian 2018 ASFV outbreak was aligned with other ASFV nucleotide sequences representing the 24 ASFV *B646L* (p72) genotypes (6, 11, 15) using the ClustalW algorithm in MEGA X (40). ClustalW was used to perform multiple sequence alignment of nucleotide sequences of the TRS as implemented in MEGA X (40). Nucleotide sequences of the *B602L* (CVR) gene were translated using the ExPASy translation tool (https://web.expasy.org/translate/) and coded in order to obtain corresponding amino acid tetramer signatures as previously described (21, 23, 29). The evolutionary history of ASFV was inferred by the maximum likelihood method using the Kimura two-parameter model implemented in MEGA X (40). Phylogeny was inferred following 1,000 bootstrap replications.

**Table 1:** African swine fever virus (ASFV) isolates from Eastern and Southern Africa used for the construction of phylogenetic tree based on partial *B646L* (p72) gene nucleotide sequences.

**Isolate**	**Host species**	**Year of isolation**	**Location**	**Country**	**Accession number**	**p72 genotype**	**Reference**
DRC/35/10/5	Domestic pig	2010	NK^a^	DRC^b^	KX121552	I	(30)
TAN/12/Iringa	Domestic pig	2012	Iringa	Tanzania	KF834193	II	(31)
BOT/1/99	Domestic pig	1999	NK	Botswana	AF504886	III	(27)
RSA/1/99/W	NK	1999	NK	South Africa	AF449477	IV	(27)
Tengani	Warthog	NK	Tengani	Malawi	AF301541	V	(32)
SPEC265	Domestic pig	1994	NK	Mozambique	AF270710	VI	(32)
RSA/1/98	NK	1998	NK	South Africa	AF302818	VII	(27)
MOZ/1/98	Domestic pig	1998	Tete	Mozambique	AF270705	VIII	(27)
Ug12.Kabale1	Domestic pig	2012	Kabale	Uganda	KC990890	IX	(33)
BUR/18/Rutana	Domestic Pig	2018	Rutana	Burundi	MK829709	X	This study
Kenya 1950	Domestic pig	1950	NK	Kenya	AY261360	X	(34)
TAN/Kwh12	Warthog	1968	Kirawira	Tanzania	AF301546	X	(27)
KAB 94/1	Domestic pig	1994	NK	Kenya	AY972163	X	(35)
KIRT/893	Ticks	1989	Kirawira	Tanzania	AY351512	X	(5)
TAN/16/Ngara	Domestic pig	2016	Ngara	Tanzania	MF437293	X	(36)
TAN/15/Mwanza	Domestic pig	2015	Mwanza	Tanzania	MF437291	X	(36)
BUR/1/84	Domestic pig	1984	Gitega	Burundi	AF449463	X	(27)
BUR/90/1	Domestic pig	1990	Muyinga	Burundi	AF449472	X	(5)
Ken05/Tk1	Tick	2005	Kapiti plains	Kenya	NC_044945	X	(34)
KAB/62	Ticks	1983	Livingstone Game Park	Zambia	AY351522	XI	(5)
MZI/921	Domestic pig	1992	Mzinda	Malawi	AY351543	XII	(5)
SUM/1411	Ticks		Sumbu Park	Zambia	AY351542	XIII	(5)
DRC/35/10/3	Domestic pig	2010	Ngaliema	DRC	KX121550	XIV	(30)
TAN/08/Mazimbu	Domestic pig	2008	Mazimbu	Tanzania	GQ410765	XV	(37)
TAN/2003/1	Domestic pig	2003	Arusha	Tanzania	AY494550	XVI	(5)
ZIM/92/1	Domestic pig	1992	Gweru	Zimbabwe	DQ250119	XVII	(38)
NAM/1/95	NK	1995	Windhoek	Namibia	DQ250122	XVIII	(38)
SPEC/251	NK	1996	Ellisras	South Africa	DQ250118	XIX	(38)
Lillie	Domestic pig	NK	NK	South Africa	DQ250109	XX	(38)
RSA/1/96	NK	1996	Gravelotte	South Africa	DQ250125	XXI	(38)
SPEC/245	NK	NK	Louis Trichardt	South Africa	DQ250117	XXII	(38)
ETH/5a	Domestic pig	2011	Bahir Dar	Ethiopia	KT795361	XXIII	(4)
MOZ_11/2006	Tick	2006	Gorongosa National Park	Mozambique	KY353990	XXIV	(6)

a *Not known*.

b *Democratic Republic of the Congo*.

**Table 2:** Tetramer amino acid repeat signatures within the central variable region (CVR) of the *B604L* gene of selected ASFV strains belonging to p72 genotype X from some East African countries.

**Strain name**	**Year of collection**	**Country of origin**	**Host**	**CVR accession number**	**CVR signature**	**Reference**
BUR/18/Rutana	2018	Burundi	Domestic pig	MT550685	AAABNAABA	This study
TAN/16/Ngara	2016	Tanzania	Domestic pig	MT550686	AAABNAABA	This study
TAN/13/Arusha	2013	Tanzania	Domestic pig	KF706367	BNBA(BN)_5_NA	(23)
Ken05/Tk3	2005	Kenya	Tick	HM745290	AAANAABBA	(39)
TAN/13/Moshi	2013	Tanzania	Domestic pig	KF706364	BNBA(BN)_5_NA	(23)
Ken08BP/HB	2008	Kenya	Bushpig	JN590917	AAABNAAAABA	Unpublished
Bur90/1	1990	Burundi	Domestic pig	AM259424	AAABNAAAAAAAAAABA	(21)
Bur84/2	1984	Burundi	Domestic pig	AM259423	AAABNAAAAAAAAAABA	(21)
Bur84/1	1984	Burundi	Domestic pig	AM259422	AAABNAAAAAAAAAABA	(21)

*Key: (CAST, CVST, CTST, CASI = A), (CADT, CADI, CTDT, CAGT, CVDT = B), (NVDT, NVGT, NVDI = N), and (CASM = D)*.

## Results

### Outbreak Description

The outbreak described in this study was reported in Mutwana village (Giharo district) in August 2018 (Figure 1). Afterward, ASF spread from Mutwana to neighboring villages of the Muzye, Butezi, Giharo, and Gakungu zones in the Giharo district before it was reported in villages of the Kinzanza and Gitanga zones of the Gitanga district in September 2018 (Figure 1). A total of 3,509 domestic pigs from 1,958 households died in both districts of the Rutana region, South-Eastern Burundi. The main clinical signs presented by affected domestic pigs included anorexia, dyspnea, and congestion of the skin particularly on the peripheral part of the pinna, belly, neck region, and mammary glands, followed by sudden death. Postmortem findings included hydrothorax, splenomegaly, and hemorrhages in the lung, liver, and lymph nodes, especially the hepatogastric and mesenteric lymph nodes.

### Confirmation of ASF Using PCR

Each of the lung, liver, and spleen obtained from pigs that naturally died from the disease were tested for the presence of ASFV as previously described (26). All lung, liver, and spleen tissues from the three sampled domestic pigs were found to be positive for ASFV. The spleen had a high ASFV DNA concentration on a nanodrop spectrophotometer, followed by the lung and liver, at 501, 336.5, and 141.5 ng/μL, respectively.

### Molecular Characterization of ASFV

**Figure 2:**
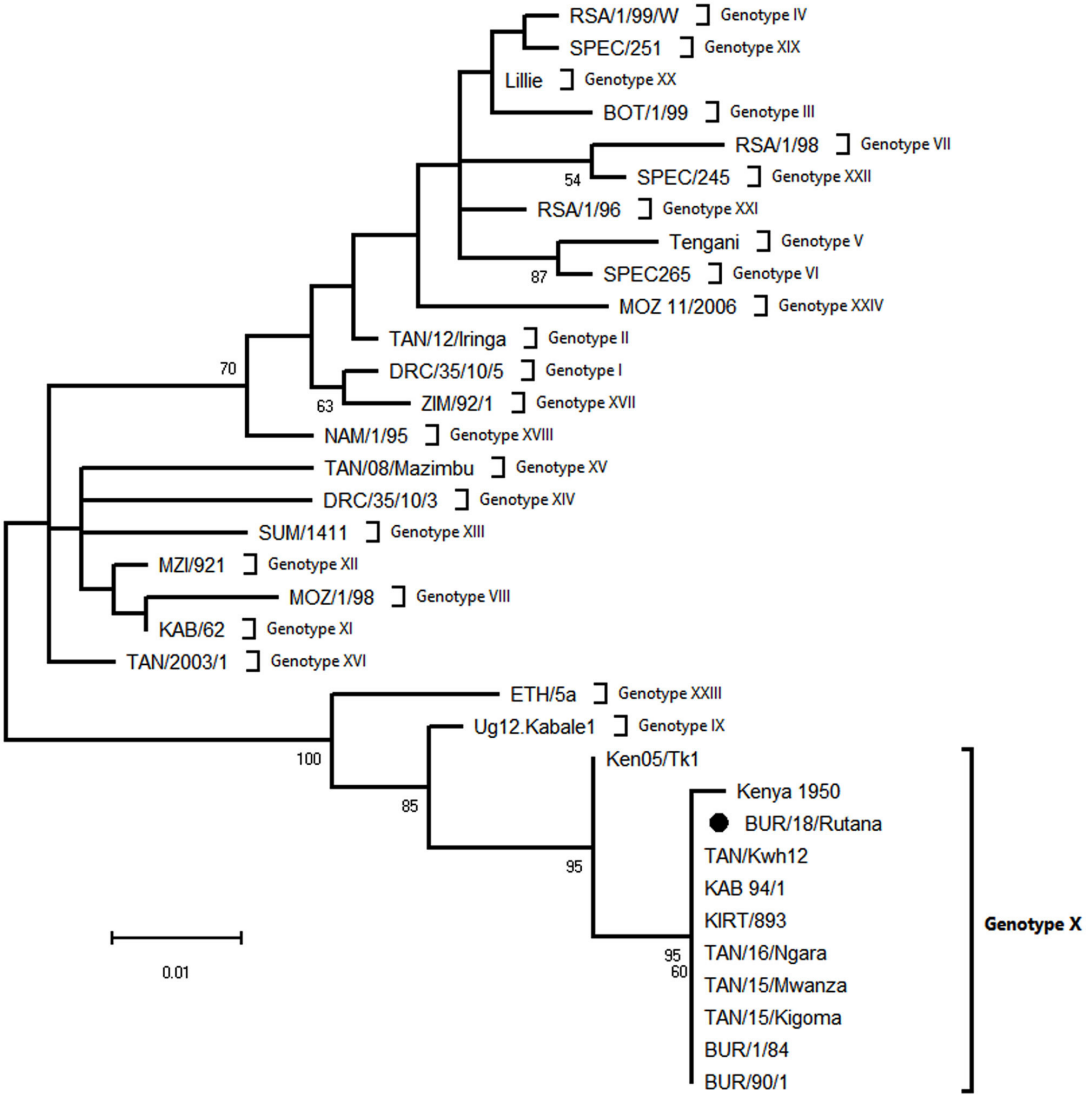
Evolutionary relationships of representative strains of African swine fever virus (ASFV) based on the maximum likelihood phylogeny of the partial p72 gene nucleotide sequences. The phylogenetic analysis was performed using MEGA X (http://www.megasoftware.net) and the Kimura two-parameter substitution model, as determined by a model selection analysis. Phylogeny was inferred following 1,000 bootstrap replications, and the node values show percentage bootstrap support (only values above 50% are shown). The round black spot indicates the ASFV nucleotide sequence from Burundi obtained in this study. The scale bar indicates nucleotide substitutions per site.

The ASFV strain from the Rutana region (South-Eastern Burundi) obtained in this study was designated as BUR/18/Rutana. BLASTn of BUR/18/Rutana *B646L* (p72) ASFV nucleotide sequences in GenBank showed high nucleotide identity to *B646L* (p72) genotype X ASFV strains previously described in Tanzania and Kenya. In order to determine the genetic relationship of BUR/18/Rutana with other ASFVs representing the 24 *B646L* (p72) ASFV genotypes, a phylogenetic tree was constructed with the maximum likelihood method using partial *B646L* (p72) nucleotide sequences. The BUR/18/Rutana ASFV strains clustered together with genotype X strains previously described in Burundi, Tanzania, and Kenya (Figure 2).

**Figure 3:**
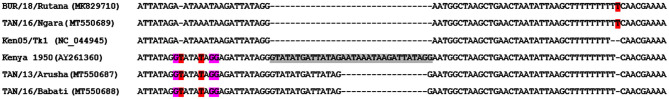
Partial nucleotide sequence alignment of the intergenic region between *I73R* and *I329L* genes in ASFV isolates belonging to *B646L* (p72) genotype X from Eastern Africa. The nucleotides highlighted in gray, present in the reference ASFV strain, are absent in the 2016 Tanzanian ASFV, the 2018 Burundian ASFV, and the tick strain described in Kenya in 2005. Other nucleotide variations between ASFV strains are highlighted in red and pink. The GenBank accession numbers of the nucleotide sequences are shown in parentheses.

We amplified the region located between the *I73R* and *I329L* genes, characterized by the presence of TRS. The most similar TRS was that of TAN/16/Ngara responsible for the 2016 ASF outbreak in domestic pigs in the Ngara district of Kagera Region, South-Western Tanzania, followed by that of Ken05/Tk1 collected from a tick in Kenya in 2005 (Figure 3). We compared the Kenya 1950 isolate, which is a reference for genotype X, with BUR/18/Rutana. The ASFV strain BUR/18/Rutana lacked a 32 bp fragment in the TRS (Figure 3), as was the case for the TAN/16/Ngara and Ken05/Tk1 strains (34). In addition, the Burundian ASFV strain described in this study had the signature AAABNAABA at the *B602L* (CVR) gene and showed 100% amino acid sequence identity to TAN/16/Ngara (Table 2).

## Discussion

**Figure 4:**
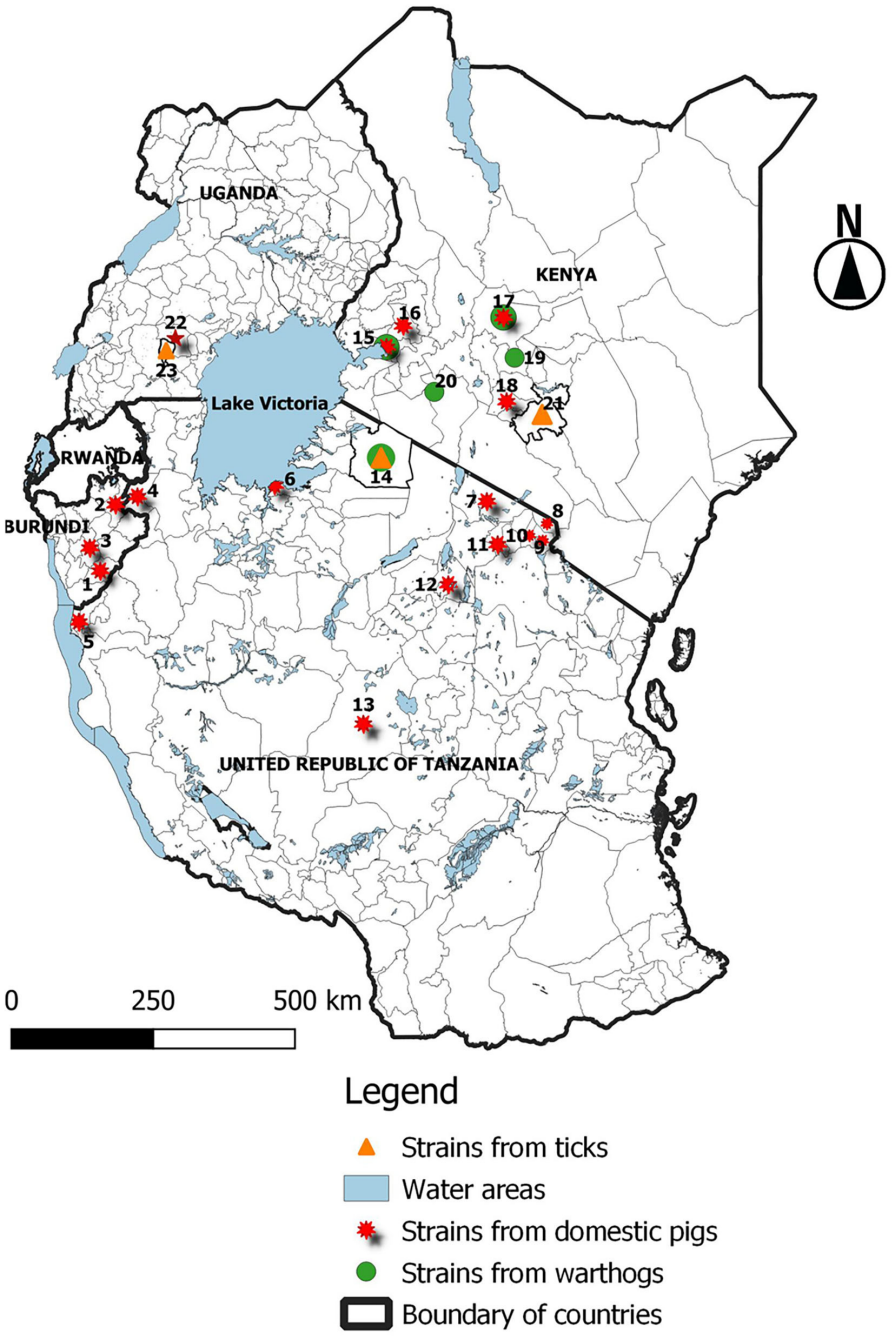
Distribution of ASFV p72 genotype X isolates in the East African Community (EAC) between 1954 and 2018. ^1^Rutana 2018 [domestic pig (Dp)]; ^2^Muyinga 1990 (Dp); ^3^Gitega 1984 (Dp); ^4^Ngara 2016 (Dp); ^5^Kigoma 2004 and 2015 (Dp); ^6^Mwanza 2015 (Dp); ^7^Longido 2009 (Dp); ^8^Rombo 2013 (Dp); ^9^Moshi 2013 (Dp); ^10^Machame 2013 (Dp); ^11^Arusha 2013 (Dp); ^12^Babati 2016 (Dp); ^13^Manyoni 2015 (Dp); ^14^Kirawira 1968 [warthog (Wh)] and 1989 [Wh and tick (Tk)]; ^15^Nyanza 2008 (Dp and Wh); ^16^Nandi 2005 (Dp); ^17^Nanyuki 1954 (Dp), 1959 (Dp and Wh), and 1961 (Dp); ^18^Kiambu 2005 (Dp); ^19^Kiganjo and Mweiga 1957 and 1959 (Wh); ^20^Rift valley 1959 (Wh); ^21^Machakos 2005 and 2009 (Tk); ^22^SSembabule 1995 (Dp); ^23^Lake Mburu national park 2010 (Tk). The isolates from Kenya in 1950 (Kenya 1950) and Uganda in 1964 (Ug64) are not indicated on the map, because their locations are not mentioned in the available literature.

ASF is endemic in Burundi, and 24,696 ASF cases have been reported in the country between January 2005 and December 2018 (13); the disease continues to economically impact on small-scale farmers. In this study, we report an outbreak of a highly fatal hemorrhagic disease of domestic pigs that occurred in 2018 in the Rutana region of Burundi. The presence of ASFV in domestic pigs was confirmed by nucleotide amplification, sequencing, and phylogenetic reconstruction of the ASFV *B646L* (p72) gene, the region located between the *I73R* and *I329L* genes characterized by the presence of TRS, and the *B602L* (CVR) gene. Partial sequencing of the *B646L* (p72) gene is used in order to determine the ASFV genotype. However, to achieve more resolution among closely related strains, analysis of additional ASFV genomic regions is needed (21, 28). Regions with tandem repeat arrays within the coding or in intergenic regions identified in the ASFV genome have proven useful for discerning between closely related ASFV strains (21). Among these regions, the TRS located in the CVR within the *B602L* gene and the TRS located in the intergenic region between the *I73R* and *I329L* genes have been described as suitable to distinguish between closely related ASFV strains and to trace the source of ASF outbreaks (21, 28, 41, 42). Thus, in the present study, *B646L* (p72), *B602L*, and the TRS between the *I73R* and *I329L* genes were analyzed to achieve higher resolution. The results obtained from the present study confirm an ASF outbreak in the Rutana region in South-Eastern Burundi. The ASFV responsible for the 2018 outbreak in the Rutana region belonged to *B646L* (p72) genotype X and was closely related to other genotype X strains previously characterized in Burundi, Tanzania, and Kenya (28, 34, 41). Genotype X is one of the predominant ASFV p72 genotypes in countries of the East African Community (Figure 4), and it has been isolated from domestic pigs, warthogs, and *Ornothodoros* ticks in the region (23, 39). The ASFV p72 genotype X has been involved in previous outbreaks in Burundi in 1984 and 1990, in Gitega and Muyinga, respectively (5, 21). The ASFV BUR/18/Rutana lacked a 32 bp fragment within TRS compared to the reference genotype X isolate, Kenya 1950 (40). Similarly, the same 32 bp fragment was absent in the ASFV TAN/16/Ngara strain responsible for the ASF outbreak in domestic pigs in South-Western Tanzania in 2016 and the ASFV Ken05/Tk1 strain recovered from a tick that was extracted from a warthog burrow in central Kenya in 2005 (40). In addition, the amino acid tetramer repeats within the CVR of the virus that caused the 2018 ASF outbreak in the Rutana region had the signature AAABNAABA and showed 100% similarity to the virus recovered from the outbreak in Ngara, South-Western Tanzania, in 2016. The amino acid identity was greater with TAN/16/Ngara than with the ASFV strains responsible for earlier outbreaks in Burundi in 1984 and 1990 (21).

The high genetic similarity of the virus described in this study to ASFV strains recovered from domestic pigs, warthogs, and *Ornithodoros* soft ticks vectors is in agreement with previous studies that classified the ASFV p72 genotype X as a sylvatic cycle associated genotype (23, 39, 43). In Burundi, the Ruvubu and Kibira National Parks host warthogs (*Phacochoerus aethiopicus*) and bushpigs (*Potamochoerus porcus*) (18), which are natural reservoirs of ASFV, but the role of the sylvatic cycle in the maintenance and transmission of ASF in the country has not been investigated. Therefore, there is a lack of information on the potential existence of the ASF sylvatic cycle in Burundi, and this aspect should be investigated in wildlife protected areas of Burundi in order to understand the possibility of the virus spilling over from the sylvatic to the domestic cycle. The strain described in this study showed high genetic similarities with ASFV strains previously reported in Burundi and those circulating in the region, indicating regional distribution and circulation of this ASFV genotype. These findings are in agreement with previous studies in the region that have also reported transboundary distribution of different ASFV genotypes including genotype X between the Democratic Republic of the Congo (DRC) and Burundi (44) and genotype II between Malawi, Tanzania, and Zambia (45–48). In these studies, uncontrolled movements of domestic pigs and pork products have been cited as a major factor contributing to the transboundary spread of ASFV strains. Sequence analysis of the three ASFV genomic regions considered in this study showed that the most closely related strain was that responsible for the 2016 ASF outbreak in the Ngara district of Kagera region, South-Western Tanzania, indicating that the same viruses are causing outbreaks on both sides of the Burundi-Tanzania border. Kagera region on the Tanzanian side and the Rutana region in Burundi share borders, and uncontrolled animal movement, including that of domestic pigs, are more likely to happen between these two regions. For instance, movement of refugees together with their livestock, reported in the area (49), can contribute to the spread of animal diseases, including ASF. It has been reported that in order to reduce the economic loss due to ASF outbreaks, some farmers sell their pigs before they show clinical signs as soon as ASF is suspected. This emergency pig sell contributes to the spread of the virus in resource-poor settings, including between countries (50–52). However, considering the proximity of the Rutana region to Ruvubu National Park, where warthogs are present (20), and the reported uncontrolled movement of wild animal species between Ruvubu National Park in Burundi, Akagera National Park in Rwanda, and the Kimisi and Burigi game reserves in Tanzania (49), the virus spillover from the sylvatic to the domestic cycle cannot be excluded based on the results of this study.

This study confirms that the 2018 ASF outbreak in the Rutana region, South-Eastern Burundi, was caused by the ASFV p72 genotype X. The virus showed high genetic similarities with ASFV strains previously described in domestic pigs, warthogs, and soft ticks in East African countries, indicating a possible common wild source and continuous circulation in domestic pigs in the region. This study contributes to the understanding of ASFV epidemiology in Burundi and in the East African Community. It will be interesting to investigate the role of the ASFV sylvatic cycle in Burundi and to perform whole genome sequencing of the ASFV strains reported in this study along with those previously described in Burundi and ASFV strains from neighboring countries to facilitate a better understanding of ASFV dynamics and epidemiology in Eastern and Southern Africa. Such perspective on the changing dynamics may provide an understanding of the global epidemiology of ASF.

## Data Availability Statement

The datasets generated for this study can be found in online repositories. The names of the repository/repositories and accession number(s) can be found in the article.

## Author Contributions

JH, GM, and JN designed the study. JH and LN participated in sample collection and laboratory analysis. JH, GM, and CY analyzed and interpreted data. JH wrote the first draft of the manuscript. JN, LN, CY, OK, DN, GM, and HN reviewed and edited the manuscript. All authors read and approved the final manuscript. All authors contributed to the article and approved the submitted version.

## Conflict of Interest

The authors declare that the research was conducted in the absence of any commercial or financial relationships that could be construed as a potential conflict of interest.
